# Peer mentoring for eating disorders: evaluation of a pilot program

**DOI:** 10.1186/s40814-018-0268-6

**Published:** 2018-04-18

**Authors:** Jennifer Beveridge, Andrea Phillipou, Kelly Edwards, Alice Hobday, Krissy Hilton, Cathy Wyett, Anna Saw, Georgia Graham, David Castle, Leah Brennan, Philippa Harrison, Rebecca de Gier, Narelle Warren, Freya Hanly, Benjamin Torrens-Witherow, J. Richard Newton, Steering group committee, Jennifer Beveridge, Kelly Edwards, Alice Hobday, Cathy Wyett, Anna Saw, Philippa Harrison, Rebecca de Gier, Richard Newon

**Affiliations:** 1 Eating Disorders Victoria, Melbourne, Australia; 2 Centre for Mental Health, Swinburne University of Technology, Hawthorn, PO Box 218 (H99), Melbourne, Victoria 3122 Australia; 3 Department of Psychiatry, The University of Melbourne, Melbourne, Australia; 4 Department of Mental Health, St Vincent’s Hospital, Melbourne, Australia; 5 Department of Mental Health, Austin Health, Melbourne, Australia; 6 School of Psychology, Australian Catholic University, Melbourne, Australia; 7 School of Social Sciences, Monash University, Melbourne, Australia; 8 Peninsula Mental Health Service, Peninsula Health, Melbourne, Australia

**Keywords:** Eating disorders, Peer mentor, Quality of life, Treatment, Peer work

## Abstract

**Background:**

Eating disorders are serious psychiatric illnesses that are often associated with poor quality of life and low long-term recovery rates. Peer mentor programs have been found to improve psychiatric symptoms and quality of life in other mental illnesses, and a small number of studies have suggested that eating disorder patients may benefit from such programs. The aim of this study is to assess the efficacy of a peer mentor program for individuals with eating disorders in terms of improving symptomatology and quality of life.

**Methods:**

Up to 30 individuals with a past history of an eating disorder will be recruited to mentor 30 individuals with a current eating disorder. Mentoring will involve 13 sessions (held approximately every 2 weeks), of up to 3 h each, over 6 months.

**Discussion:**

This pilot proof-of-concept feasibility study will inform the efficacy of a peer mentoring program on improving eating disorder symptomatology and quality of life, and will inform future randomised controlled trials.

**Trial registration:**

Australian and New Zealand Clinical Trials Registration Number: ACTRN12617001412325. The date of registration (retrospective): 05/10/2017.

**Electronic supplementary material:**

The online version of this article (10.1186/s40814-018-0268-6) contains supplementary material, which is available to authorized users.

## Background

Eating disorders are serious psychiatric conditions with high rates of morbidity and mortality. They are associated with a staggering socioeconomic cost of almost $70 billion per year in Australia alone [1]. Anorexia nervosa is associated with the most serious consequences, having the highest death rate of any mental illness, with one in ten of those diagnosed dying as a result of the physical effects of starvation or suicide [2, 3]. The efficacy of treatments for eating disorders is limited, often resulting in suboptimal recovery and high relapse rates. Anorexia nervosa, specifically, is associated with long-term recovery rates of less than 50% among surviving patients [4, 5]. The complex nature of eating disorders demands an innovative and specialised solution to positively impact on relapse and recovery rates. Research has shown that maintaining social connections and applying life skills can support recovery in eating disorders and other mental illnesses (e.g. [6, 7]); yet, little research has been undertaken in the eating disorders field, particularly in relation to peer support programs.

Peer support is based on the belief that people who have faced, endured and overcome adversity can offer useful support, encouragement, hope and mentorship to others facing similar situations [8]. In broad terms, peer work can be classified into three categories [9]: self-help which involves voluntary peer support, self-referral and often independent and autonomous groups; consumer operated which refers to establishments where consumers run the organisation; and integrated which involves either voluntary or paid peer work that is positioned within existing mental health or related organisations and services. Most peer work in Australia sits within an ‘integrated’ category, where peer workers operate within existing mental health services, often working as team members alongside mental health workers.

Mental health peer work is a relatively new approach to service delivery. Evaluation has lagged behind implementation of peer workforce roles; however, a number of randomised controlled trial findings are available, and although the evidence base is a developing one, there is a strong indication that a substantial peer workforce in a psychiatric service will improve patient outcomes [10]. Peer mentor programs, which encompass support programs delivered by individuals typically in recovery from an illness to those with a current diagnosis, have been found to reduce hospital readmission rates in a range of mental health issues. For example, a study by Sledge et al. [6] utilised a mixed sample of psychiatric patients and found that those assigned a peer mentor had significantly fewer re-hospitalisations and shorter stays in hospital than patients who did not participate in the program. It has been noted in the literature that peer workers themselves can be both positively and negatively affected by providing peer support. This is an important and underexplored area. In a study specifically examining the impact of mentoring programs on people with eating disorders, Perez et al. [7] reported improved quality of life and family relationships, and improvements in psychological, emotional and physical wellbeing in individuals matched with a mentor. In this program Mentor Connect, the mentor-mentee relationship aimed at supporting the mentee to try new skills and work towards recovery-based goals [7]. Cardi et al. have a trial in progress at present assessing the use of peer support in the SHARED (Self-Help And Recovery guide for Eating Disorders) trial [11]. Other than these early evaluations, very little evidence is yet available in the literature to support peer mentoring in eating disorders [12].

A recent systematic review and synthesis of the impact of peer support on eating disorders yielded only four eligible studies (*N* = 270*)* [13]. One such study, which evaluated a mentoring program for women with eating disorders [7], reported that quality of life and treatment compliance were significantly increased in matched (*n* = 58) compared to unmatched (*n* = 49) mentees. In another study, an 8-week mentoring pilot program for undiagnosed (subclinical) adolescent girls (*n* = 31) demonstrated a significant reduction in disordered eating by the conclusion of the program [14]. In a qualitative study included in the review [15], mentees reported feeling understood, ‘normal’ and inspired, as a result of sharing with someone who had been through the same experience. A major theme that Fogarty et al. [13] identified from the synthesis of the above studies was the sense of belonging mentees experienced through the development of the mentoring relationship. Developing supportive relationships and an increased sense of belonging have been identified as an important aspect of eating disorder treatment [16], and these factors have been found to impact positively upon motivation for recovery [17]. Indeed, one study reported that recovered individuals attributed much of their recovery to aspects outside of formal therapy and placed particular emphasis on interpersonal relationships [18].

Potential benefits to the mentees’ recovery process notwithstanding, the feasibility of the current peer mentoring program relies on the delivery of mentoring that is sustainable and non-maleficent from the point of view of the mentors. It is therefore important to examine and analyse the likely risks and benefits associated with the provision of mentoring from this perspective, as these individuals are likely to have vulnerabilities associated with their previous diagnosis [19]. The peer mentoring literature in the broader mental health domain has identified several benefits that exist for mentors, including increased confidence, validation of their own recovery [20] and stable employment [21, 22]. However, it is unclear whether these benefits can be generalised to individuals who have recovered from an eating disorder.

There is little available research on the evaluation of risks to recovered individuals working in a mental health peer support role. In the literature that does exist, boundary issues and transgressions are cited as common challenges encountered by mentors [23–25]. Specifically, issues of confidentiality, multiple relationships, role confusion [26] and relational vulnerability due to self-disclosure [19, 25] have been identified as potential risks to the wellbeing of mentors. In a qualitative study investigating benefits and limitations of employing recovered individuals to deliver mental health services, mentors commonly reported they experienced stress associated with mentoring unmotivated or uncooperative mentees and highlighted the importance of adequate supervision and training [22]. Finally, the risk of relapse is important to examine, because if a relapse of an active mentor occurred, it would not only be (evidently) detrimental to the mentor, but it could also undermine the process of instilling hope in the mentee [25].

The examination of potential risks and benefits to mentors in this particular pilot program through both qualitative and quantitative data collection methods will attempt to assess whether the recruitment, training and support currently in place are effective and sufficient to ensure the wellbeing of the mentors, which is of paramount importance when considering the long-term feasibility of the program.

Previous peer mentoring programs in Victoria, Australia, have focused on a range of mental illnesses, though none have focused on people with eating disorders to date. In this context, we describe here a peer support program for people with an eating disorder, aimed at addressing a service gap in Victoria, Australia, for those who require additional support to sustain recovery while living independently.

### Study AIMS

The aim of this study is to determine the efficacy of a peer mentor program for people who have received eating disorder treatment in a specialist hospital setting or equivalent, in improving eating disorder symptomatology and quality of life, to inform future randomised controlled trials. An additional aim is to determine the feasibility of the study by assessing participants’ capability to complete the mentoring sessions and the program. The study’s feasibility will be measured in terms of having a sufficient number of participants sign up, participate and be retained until the end of the study and also that they tolerate and accept the intervention, that the intervention can be delivered as per protocol and that there are no concerning adverse events related to the study. The current program is a new program that combines peer support principles with documented evidence, balanced against available resource and organisational risk constraints. The program is designed to harness the experience of people who have recovered from eating disorders and combine it with regular de-briefing and supervision. This project aims to ensure eating disorder patients leaving hospital can better sustain their recovery outcomes in the long term. This is the first time such a program has been offered in Victoria, Australia. Thus, the primary aim of the study is to evaluate the effectiveness of the peer mentor program on eating disorder patient outcomes. A secondary, exploratory aim of the program is to evaluate the effects of the program on peer mentors in terms of eating disorder symptomatology and quality of life.

### Primary hypotheses

Participation in the peer mentor program is hypothesised to improve eating disorder symptomatology and quality of life, and reduce relapse rates in eating disorder participants (i.e. ‘mentees’, also referred to as ‘participants’ in appendices).

## Methods/design

### Study design and mentor-mentee matching

The study is a multi-site collaborative trial conducted at Eating Disorders Victoria (EDV), The Melbourne Clinic (TMC),  and the Body Image and Eating Disorders Treatment and Recovery Service (BETRS) at the Austin Hospital (inpatient unit) and St Vincent’s Hospital (intensive day patient program), all in Melbourne, Victoria, Australia. Up to 30 mentees with an eating disorder (anorexia nervosa, bulimia nervosa, binge eating disorder or eating disorder not otherwise specified, according to the Diagnostic and Statistical Manual of Mental Disorders 5) will be matched with up to 30 mentors with a past history of an eating disorder. Participation will involve a longitudinal study assessing the efficacy of a peer mentor program for individuals with eating disorders. All mentees and mentors will be over the age of 18 years.

As this is a pilot proof-of-concept feasibility study, a randomised controlled trial will not be undertaken, but the program will be evaluated with a within-group design. Participation will involve a series of 13 mentoring sessions of up to 3 h each (ideally, every 2 weeks) over 6 months, following the mentee’s discharge from the BETRS/TMC inpatient unit or transition to/from the BETRS/TMC intensive eating disorders day program. The peer mentoring program will be delivered in a variety of community settings, i.e. local shopping centres, parks and cafes. Mentees and mentors will not be permitted to meet in either person’s home.

Mentors will be employed as ‘casual’ employees of EDV (see Additional file 1: Appendix A) and will be reimbursed for any reasonable expenses incurred during mentoring sessions as part of their employment, i.e. lunch for mentee and mentor, public transport fares for mentee and mentor practicing independent use of public transport, etc. Mentors and mentees will not receive any payment or reimbursement for this research.

Mentees and mentors will be paired on the basis of information provided by both parties. Mentees will be asked to provide a short statement about their values and interests and information about their preferences for working with a mentor of a same/different age, gender, eating disorder history and geographic area. Mentors will be asked to provide a short personal statement about their values and work style to facilitate effective matching. Each pair will be matched with a focus on the best match for the mentee by EDV and BETRS/TMC staff. If a mentor chooses to discontinue their employment on this project, EDV will re-match the mentee with another mentor as soon as practicable and they will continue to work towards the goals identified in their Wellness Plan (see Additional file 1: Appendix B). If a mentee chooses to discontinue with the program, then their mentor will return to the pool of mentors available to be matched with another referral.

### Recruitment procedure

Mentees will be recruited from the partner hospital inpatient unit (the Austin Hospital) and intensive day program (St Vincent’s Hospital), collectively known as the BETRS; and TMC. A member of the research team at BETRS/TMC will identify potential mentees and approach them with the study information form. Mentors will be recruited by EDV according to EDV policy and procedures. Mentors will be employed explicitly for this role and will be invited to participate in the research component of the program by one of the research staff. Participation will be voluntary. The suitability of mentors will be assessed during the interview process for their employment and by one of the researchers when approached for participation in the research component of the project. A minimum of 2 years in recovery will be required as indicated by the clinical measures that will be used to assess current or recovered eating disorder stages (demographics and medical history form).

Written informed consent will be obtained from all participants (mentees and mentors) prior to the commencement of the peer mentor program, by a member of the research team.

#### Eligibility criteria

Inclusion criteria for *mentees* include:

- Transitioning out of the BETRS inpatient program at the Austin Hospital or TMC, or transition in/out of the BETRS/TMC outpatient/day patient program
- Current diagnosis of an eating disorder (anorexia nervosa, bulimia nervosa, binge eating disorder or eating disorder not otherwise specified) according to the Diagnostic and Statistical Manual of Mental Disorders (DSM-5)
- Currently actively receiving treatment for their eating disorder for the duration of the program. (See Wellness Plan in Additional file 1: Appendix B). At a minimum, mentees must be receiving care from a GP and a psychologist.

Inclusion criteria for *mentors* include:

- Recovery from an eating disorder for a minimum of 2 years as indicated on the demographics and medical history form, in combination with the Eating Disorder Examination Questionnaire (EDE-Q). The wellness criteria are evidenced by self-reported absence of eating disorder symptoms (indexed by the EDE-Q), being weight-restored, not receiving treatment for eating disorders symptoms and use of a personal Wellness Plan/self-care activities to maintain their recovery. The Wellness Plan also outlines signs that indicate a mentor is struggling and how to best support them during these times.
- Successful recruitment as a staff member at EDV, including referee checks and agreement code of conduct

Exclusion criteria for mentors and mentees include anyone who is at serious risk of harm to oneself or another, as determined by the clinical team, for example, individuals at high risk of suicide.

### Mentor training

Mentors will attend a 3-day training and induction program prior to the program rollout to ensure that mentors are prepared in their role. EDV staff and external trainers deliver different elements of the training dependent on their experience. As the mentors are EDV staff, the training starts with inducting them as employees and going through the procedures and policies outlined for all EDV staff members.

An overview of the Intentional Peer Support (IPS) model is given to all mentors as a framework to base all interactions. This model states that peer support is intentional, in that we come to the relationship with a specific purpose in mind—with the program, this is maintaining recovery. The four core elements of IPS are connection—between the mentor and mentee, holding a worldview and understanding how we have come to know what we know and mutuality—learning and growing together and moving towards identified goals (recorded in the mentee’s Wellness Plans) [27].

Training also covers off the challenging situations mentors might be faced with when supporting their mentee. Information about the program’s aims and objectives are made clear so the mentors know what they are working towards. As there is a high possibility of self-harm and thoughts of suicide, all mentors complete an internationally recognised safeTALK suicide alertness workshop which covers hands-on skills to be able to respond to someone who has the thoughts of suicide [28]. The EDV psychologists also facilitate role-plays with mentors which raise common issues and are discussed.

### Mentoring activities

The program will involve 13 mentoring sessions of up to 3 h each, ideally completed every 2 weeks, over 6 months. Mentoring activities will include peer support activities such as providing information, emotional support and sharing of their own recovery experience. Mentoring activities are guided by each individualised mentee’s Wellness Plan (see Additional file 1: Appendix B), formulated during the first mentoring session. This focuses on the mentee’s short-term goals in the following domains: living circumstances and skills, health, self-care, social relationships and connectedness, creative interests and hobbies, work/career and education, identity and sense of self, and community roles and responsibilities. The Wellness Plan also outlines the mentee’s current recovery strategies (e.g. thoughts, feelings, behaviours, characteristics, personal qualities, interests, activities and relationships) that support the mentee to remain healthy as well as articulate warning signs that indicate additional professional support or treatment should be sought (e.g. symptoms, thoughts, images, feelings, mood and behaviours). Mentors will also complete their own amended Wellness Plan which allows them to document self-care strategies and identify potential risks to ensure that program staff can support and ensure the mentor role is not detrimental to their continued health and wellbeing.

Sessions 2–12 of the mentoring activities will focus on supporting the mentee to work toward their identified goals through community-based activities such as supermarket shopping, self-care activities, reconnecting with community or navigating social interactions involving food. Mentors will share aspects of their own recovery stories to promote hope and provide an empathic response to the challenges associated with recovery.

Session 13 of the mentoring activities will focus on completion of a Program Summary outlining the mentee’s progress and achievements (see Additional file 1: Appendix D). This will include a future-focused component encouraging the mentee to identify strategies and alternative supports to draw upon after the pilot program has concluded, in order to sustain their progress.

Mentors will complete an online questionnaire at the conclusion of each mentoring session to document progress and monitor safety considerations (see Additional file 1: Appendix C). This will be reviewed by the EDV project coordinator and allows timely follow-up with any mentee or mentor needing individual support.

### Facilitated group sessions

Throughout the program, mentees will also participate in three group sessions with other mentees, facilitated by an expert clinician in eating disorder treatment. These sessions enable mentees to provide feedback on their experience of the program and increase a sense of community among mentees. Mentors will also attend three group sessions facilitated by an eating disorder clinician in order to debrief about their experiences working in the program and to increase a sense of community among mentors.

All group sessions will take place at the EDV office and will be facilitated by two professional staff, including one with psychological qualifications. The group sessions will be offered three times to mentees during the program. Group sessions for mentors will be provided three times during the program and all mentors will be expected to attend.

### Program evaluation

**Fig. 1:**
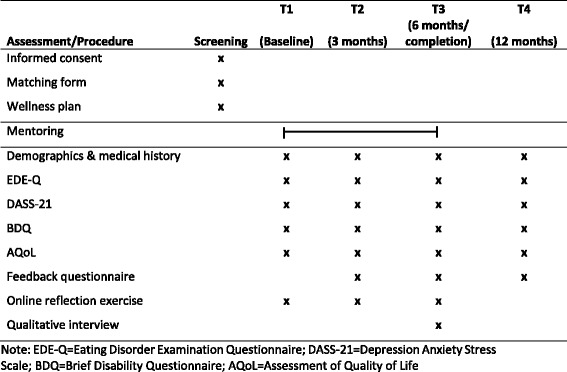
Study procedures, mentee and mentors

Informed consent will be sought by one of the research staff from all mentees and mentors to take part in the evaluation of the program, which includes quantitative and qualitative measures. Quantitative data will be collected in a re-identifiable form at baseline (beginning of the program), at 3-month (mid-program) and 6-month (end-of-program) time points, and at 12-month follow-up for both mentees and mentors (see Fig. 1). Qualitative assessments will involve online reflection exercises completed at baseline, mid-program and end-of-program time points, and a qualitative interview undertaken upon completion of the program for both mentees and mentors. In addition, attrition will be tracked by the researchers through records of sessions that the mentors keep.

#### Quantitative assessments

The following quantitative measures will be collected at each time point to determine the efficacy of the peer mentor program for reducing eating disorder symptoms and quality of life:

- Clinical demographic and medical history questionnaire: Basic demographic information and a brief medical history will be collected at baseline. At other time points, changes to demographic information and medical history (e.g. new hospital admissions) will be recorded.
- Eating Disorders Examination Questionnaire (EDE-Q) [29]: The EDE-Q is a 28-item self-report measure of psychological constructs shown to be clinically relevant in individuals with eating disorders. This measure will be used to gather information about eating disorder symptomatology.
- Depression Anxiety Stress Scale (DASS-21) [30]: The DASS is a 21-item self-report instrument designed to measure the three related negative emotional states of depression, anxiety and tension/stress. Common comorbidities such as depression and anxiety [31] can impact outcomes as well as engagement and adherence and are important targets for intervention as well as being potential adverse outcomes which require monitoring and appropriate action if they reach concerning levels of risk.
- Brief Disability Questionnaire (BDQ) [32]: The BDQ is a self-report measure of disability in everyday activities and yields two main subscales: physical disability and mental health disability.
- Assessment of Quality of Life (AQoL) [33]: The AQoL is a self-report instrument that measures health-related quality of life.
- Feedback questionnaire (not at baseline): General feedback on the program will be sought.

#### Qualitative assessments

Mentees and mentors will also complete a qualitative online reflection exercise (informed by Broom et al. [34]; see Additional file 1: Appendix E) prior to commencing the program (at baseline) and at 3 months (refer to Fig. 1) to determine the efficacy and feasibility of the program. At the completion of the program (i.e. 6 months), mentees and mentors will complete a final online reflection exercise and take part in semi-structured qualitative interviews.

The aims of this aspect of the study are to investigate the experience of mentors and mentees and to identify positive and negative aspects of the experience and factors related to these positive and negative experiences. Using the six-stage approach described by Braun and Clarke [35], thematic analysis will be undertaken to identify key themes in the data gathered through online reflection exercises and qualitative interviews. Data familiarisation (stage 1) will be effected through processes of transcription (of interviews) and close reading of all qualitative data. Preliminary coding (stage 2) identifies themes and patterns discussed by mentees; this process also supports the iterative refinement of the interview schedule (discussed below). The more systematic stage 3 involves the structured identification of recurrent themes, which is followed by a process of refining the codes so that redundant codes can be removed from our analysis (stage 4). A process of cross-checking ensures that the findings of the study are coherent and rigorous. The main themes identified following these processes (stage 5) will form the foundation of the research findings, which are then disseminated (stage 6).

##### Online reflection exercises

Mentees and mentors will be emailed a link to a secure online survey at the time points outlined in Fig. 1 and will have access to this link for 10 days. Online reflection exercises completed by mentors and mentees will allow for detailed exploration of the most important, empirically relevant themes associated with participating in a mentoring program.

Online reflection exercises will cover areas A–C in Additional file 1: Appendix F, with language modified slightly to reflect the current stage of the program. For example, questions asked at baseline will be future-oriented (‘Why did you decide to participate in the mentoring program?’ or ‘What might be some of the positive aspects of participating in this program?’). For mentors, these questions will also include reflections on their experience of the recruitment and training process. Questions asked while the program is ongoing will be based on current experiences (for example, ‘What has been your experience of participating in the mentoring program so far?’). Questions asked at completion of the program will ask respondents to reflect on their experiences of the program as a whole.

##### Qualitative interviews and feasibility

Given the limitations of the existing literature, the explorative nature and the qualitative research methodology of the current investigation, a broad semi-structured interview schedule will be used on completion of the program. This will be revised iteratively throughout the study to enable exploration of new themes raised by participants (mentors and mentees) and enable detailed exploration of the most important and relevant themes associated with participating in this program. The qualitative interviews will also cover areas A–C (see Additional file 1: Appendix F). The number of mentoring sessions completed and the participants’ capability to complete the mentoring sessions will also be assessed through the qualitative interviews.

Feasibility of the program will be guided by the CONSORT statement regarding assessing feasibility of trials [36]. Specifically, feasibility will be measured in terms of achieving sufficient numbers of participants signing up, participating and being retained in the program through the qualitative interview and records kept by mentors regarding program participation. The study will be deemed feasible if 80% of participants are able to complete the program and the target sample size is achieved within 2 years. Feasibility will also be measured in terms of tolerance and acceptance of the intervention, that the intervention can be delivered as per the protocol and identifying any adverse events related to the study through the qualitative interview and the participant feedback questionnaires. Any serious adverse events will result in the intervention not continuing to an RCT.

### Risk management and safety

Though the study is not associated with any physical risk to mentees or mentors, potential psychological risk exists through participation in the program. If mentees experience distress at any time during the program, they will be advised to contact the EDV Project Coordinator or the research team as soon as possible. The research team will determine the suitability for continuing the participant with the program and will contact emergency services and/or provide information related to psychological support available, as appropriate.

Mentors will have their own Wellness Plan that outlines their current supports and contact details in case of emergency. Mentors will also complete an online survey following each session with the participant, which has facility to request a support call from the EDV team. Mentors and mentees can also contact the project coordinator or psychologist any time for individual support, and they will attend three group debriefing sessions throughout the program. There are guidelines in place to manage self-reported increased eating disorder symptoms/risk for mentees which include referring mentees back to their treating clinician for assessment and support.

All data will be securely stored via password protection (electronic data) and under lock-and-key (hard copy data) at EDV in the short term, prior to long-term storage at St Vincent’s Hospital.

### Handling of withdrawals and replacements

Participants (mentees or mentors) are free to withdraw from the study without explanation. Data collected will be retained, unless otherwise specified by the participant. If agreed by the participant, they will also be afforded the opportunity to complete relevant follow-up assessments.

If a mentee or a mentor withdraws from the program, replacement participants will be sought. This will ensure that mentees will not be significantly affected by their partner’s (mentor) withdrawal and that the study has sufficient statistical power to identify efficacy of the program. Debriefing will be offered by program staff to the remaining participant, and mentees will be re-matched with another suitable mentor.

## Statistical methods

### Sample size estimation and justification

Thirty mentors and a minimum of 30 mentees will be recruited. This minimum sample size of 60 participants will enable differences to be identified between time points in a pilot evaluation (i.e. 28 participants per group will enable the identification of a moderate effect size with power of 0.8 and alpha of 0.05 for a within-group analysis between baseline and the primary endpoint). Sample size has been determined using the qualitative principle of data saturation, which provides an estimate of sample size based upon the comprehensiveness of the data set to identify all of the issues affecting the population under study. As Fusch and Ness [37] highlight, data saturation is the point when (a) no new insights related to the research aims and objectives are gained from additional interviews or through further analysis and (b) there is sufficient information gathered for the study to be replicated. The proposed participant numbers of 60 or more participants (30 per group) is deemed be sufficient to capture the variance in information that occurs within each participant group. Guest and colleagues [38] argue that data saturation occurs after as few as six interviews, with all information (themes) fully elucidated after 12 interviews. Our prior clinical-based qualitative research [39–41] indicates that this varies considerably based on the population under study but typically occurs after 7 and 14 interviews. Given the variability in the present sample by age, gender and anything else, our planned sample will be sufficient to gather powerful information on our study aims and objectives.

### Statistical methods

Quantitative analyses will include within-group analyses of variance (ANOVAs) and non-parametric assessments between time points, for mentees and mentors separately. Multiple imputation procedures that utilise the expectation-maximisation (EM) algorithm with bootstrap estimates of standard errors will be used for missing data. Using the six-stage approach described by Braun and Clarke [35], thematic analysis will be undertaken to identify key themes in the qualitative data.

## Discussion

Eating disorders are disabling and often long-term conditions. The efficacy of available treatments is limited, resulting in high relapse rates and significant morbidity and mortality. The current trial aims to assess the efficacy and feasibility of a peer mentor program (as a supplement to standard treatment) for patients with eating disorders, in terms of improvements in both eating disorder symptomatology and quality of life, as well as relapse rates. A unique aspect of this research is to assess the impact of this program on mentors to identify the positive and/or negative impact that engaging with current eating disorder patients has on someone who has recovered from an eating disorder. This study will assess any adverse effects on mentees or mentors and glean information that will inform refinement of the intervention for a full-scale randomised controlled trial. The findings of this pilot program will inform larger randomised controlled trials assessing the efficacy of peer mentoring for individuals with eating disorders, by providing preliminary data on the need of the program (i.e. participant flow and retention rate, and the magnitude of the outcomes to be clinically significant).

### Trial status

Protocol version: 1.5, 12/12/2017. Date recruitment began: 20/01/2017. Estimated date recruitment will be completed: 30/06/2018.

### Dissemination of results

Findings will be published in peer-reviewed journals and presented in national and international conferences. No restrictions are placed on the publication of results. Author eligibility will be based on ICMJE criteria.

## Additional file

Additional file 1: Appendix A: Excerpt from peer mentor position description. Appendix B: Wellness plan. Appendix C: Online mentor questionnaire. Appendix D: Program summary. Appendix E: Online reflection exercise. Appendix F: Qualitative interview themes. (DOCX 1383 kb)

## Abbreviations

ANOVA: Analysis of variance
AQoL: Assessment of Quality of Life
BDQ: Brief Disability Questionnaire
BETRS: Body Image and Eating Disorders Treatment and Recovery Service
DASS-21: Depression Anxiety Stress Scale
DSM-5: Diagnostic and Statistical Manual for Mental Disorders
EDE-Q: Eating Disorder Examination Questionnaire
EDV: Eating Disorders Victoria
TMC: The Melbourne Clinic
