# Supplementary material for: Peer mentoring for eating disorders: evaluation of a pilot program
Source: Pilot Feasibility Stud. 2018 Apr 18;4:75. doi: 10.1186/s40814-018-0268-6 (PMC5934861; doi:10.1186/s40814-018-0268-6)
Supplement: Supplementary file 1 — Appendix A: Excerpt from peer mentor position description. Appendix B: Wellness plan. Appendix C: Online mentor questionnaire. Appendix D: Program summary. Appendix E: Online reflection exercise. Appendix F: Qualitative interview themes. (DOCX 1383 kb) [file 40814_2018_268_MOESM1_ESM.docx]

**Additional file 1**

**APPENDIX A: Excerpt From Peer Mentor Position Description**

TITLE: Peer Mentor – Peer Mentoring Program

### ABOUT EDV

**Our Vision**

A future where the incidence, duration and impacts of all eating disorders are reduced and ultimately eradicated.

**Our Mission**

The Eating Disorders Foundation of Victoria is the primary source of support, information, community education and advocacy for people with eating disorders and their families in Victoria. We connect those whose lives are affected by eating disorders with the people, services and hope they need for recovery.

**Our Values**

We embrace the following values to underpin our vision and mission:

- Respect
- Acceptance
- Hope

Eating Disorders Victoria (EDV) is a non-profit organisation, which supports those affected by eating disorders, and their families. Eating Disorders Victoria also provides information, education and advocacy about eating disorders to the wider community. The organisational values are Respect, Acceptance and Hope, and these values underpin a philosophy of recovery. The organisation is led by a Board of Management, and is funded by state government, philanthropic funds, fee for service activities and donations. Much of EDV’s work is based on the principles of Mutual Support and Self Help. Volunteers are critical members of the team, often bringing their lived experience of eating disorders to their role, and enhance the organisation’s activities in a variety of ways including assisting EDV staff to respond to requests for information, referral and support, and in the provision of support groups and community education activities.

### ROLE STATEMENT

In 2016 EDV developed a Peer Mentoring Program in partnership with one of Melbourne’s public hospitals to support adults with an eating disorder leaving hospital inpatient units and intensive day programs with an aim of sustaining their recovery outcomes in the long term. This program has now completed its first round and will continue for a second cohort of participants.

EDV Peer Mentors will be matched with a person in recovery from an eating disorder, and have regular contact with the program participant over a six-month period (13 mentoring sessions). The Peer Mentor supports the participant to work towards the self-identified goals in their Wellness Plan, which may include independent activities of daily living (e.g. supermarket shopping), further developing meaningful skills and interests, and engaging with community life (e.g. connecting with local community groups, learning a new skill or activity, applying for or returning to work). Activities of the Peer Mentoring Program will take place in a variety of community settings.

EDV Peer Mentors will also participate in the evaluation of this program through a range of activities such as completing questionnaires, interviews and focus groups throughout the program. These activities are voluntary and managed by an independent evaluation team. The decision to take part in evaluation activities is entirely independent of a Peer Mentor’s employment, and anyone who chooses to take part in the evaluation can withdraw their consent at any time without affecting their employment at EDV.

### KEY ACCOUNTABILITIES

- Support program participant/s to devise a Wellness Plan to reach their short-term goals.
- Fortnightly contact (up to 3 hours) with assigned program participant/s to assist them to work towards the goals of their Wellness Plan over a period of six months (13 mentoring sessions).
- Participate in regular group debriefing sessions facilitated by EDV staff (three sessions over six months).
- Participant in regular individual debriefing with EDV staff.
- Provide fortnightly reports including documented contacted with program participant/s and progress against identified objectives.
- Report any issues of concern to the Project Coordinator in accordance with guidance provided by EDV.
- Participate in a three-day induction/training program, and professional development activities as required.
- Other duties as directed by the Project Coordinator.

### KEY SELECTION CRITERIA

**Essential:**

- Recovery from an eating disorder.
- Ability and willingness to discuss own experiences of mental illness in the context of supporting someone who has recently undergone treatment for an eating disorder.
- Strong commitment to peer support and understanding of the value of peer work in mental health recovery.
- Understanding of the role of professional treatment in recovery from an eating disorder.
- Demonstrated understanding of professional boundaries and ability to work within specified program guidelines.
- Ability to learn from own actions, take feedback, modify behaviour in response.
- Demonstrable knowledge of eating disorders, body image and related issues; including knowledge of the risk/protective factors, treatment options, impact on families and challenges associated with recovery.
- Excellent listening abilities and communication skills.
- Validating, encouraging and friendly manner.
- Understanding of the importance of self-care and demonstrated ability to enact own self-care routines during times of stress.

**Highly desirable:**

- Qualification in Peer Work.
- Experience as peer mentor or facilitator in another role.
- Relevant tertiary qualification in Social Work, Psychology, Public Health, Social Sciences or similar.

**APPENDIX B: Wellness plan**

**APPENDIX C: Online Mentor Questionnaire**

Mentors will be required to complete a Fortnightly Mentoring Record following each Peer Mentoring session. Mentors will be followed up by a Program staff member if they do not complete the Summary within 3 days of the session.

The questions of the Mentoring Record are designed to capture information regarding:

- Session details - including date, time, duration and attendance.
- The types of activities engaged in during the session.
- Details regarding topics discussed during the session.
- Information specific to the participants identified goals in their Wellness Plan.
- Challenges faced by the Peer Mentor during the session and whether contact has been made with EDV Project staff about this.
- Aspects of the session that went well/worked well during the session from the Mentor’s perspective.
- An opportunity to communicate any further important details regarding the session.
- An opportunity to receive a follow-up call from EDV Project staff if required following the session.
- An opportunity to provide a final reflection on the session in preparation for the next session.
- Details of the next scheduled Peer Mentoring session including date and time.

**APPENDIX D: Program Summary**

******

**APPENDIX E – Online Reflection Exercise**

**ONLINE REFLECTION EXERCISE - #1**

Throughout the pilot, we are asking participants to complete regular reflections to help us understand your experience of the program. There are no right or wrong answers to these questions, and your answers may or may not change as you progress through the program. Please answer open and honestly, in as little or as much detail as you are comfortable. Your responses will be de-identified and analysed at the end of the pilot as part of our evaluation of the Peer Mentoring Program. This program has been approved by the research ethics departments of the Austin and St Vincent's Hospitals (REF# HREC/16/Austin/514; HREC 04/16). Any concerns can be directed to A/Prof Richard Newton on (03) 9496 6496 or [richard.newton@austin.org.au](mailto:richard.newton@austin.org.au), or the Austin Health Human Research Ethics Committee on (03) 9496 4090 or at [ethics@austin.org.au](mailto:ethics@austin.org.au).

A – Reasons for participating

1. Why did you decide to participate in the Peer Mentoring Program?

2. What do you think some of the positive aspects of participating in the program will be?

3. What do you think some of the negative aspects of participating in the program will be?

B – Ideas about peer support

4. What do you think the key aspects of ‘peer mentoring’ are? How would you describe peer mentoring? (or ‘peer support’- these terms are used interchangeably in this exercise)

5. How do you think peer mentoring differs from treatment?

6. What do you think peer mentoring offers that treatment does not?

C – Benefits and challenges

7. What are some of the potential benefits for participants and/or mentors?

D – Barriers to participation

8. Thinking about barriers to participation, what do you think might stop someone from taking part in the Peer Mentoring Program? (you might like to talk about barriers relating to travel distance, logistics, illness, embarrassment, shame, or anything else you think is important)

**APPENDIX F – Qualitative Interview Themes**

1. General question/s regarding the mentoring program, the decision to participate, the experience of participating, positive and negative aspects of participation. For example:

“Why did you decide to participate in the mentoring program”?

“What was your experience in participating in the mentoring program”?

“What were the positive aspects of participating in the mentoring program”?

“What were the negative aspects of participating in the mentoring program”?

1. Follow up questions regarding the following areas (areas identified in the peer mentoring literature):
2. Definition of peer support
3. How it differs from treatment
4. What it adds to treatment
5. Potential perceived benefits to mentees/mentors
   1. Recovery
   2. Readmission
   3. Social support
   4. Social functioning
   5. Empathy and acceptance
   6. Reducing stigma
   7. Increasing hope
6. Potential perceived challenges to mentees/mentors
   1. Boundaries
   2. Power
   3. Stress
   4. Accountability
   5. Maintaining the role
7. Potential barriers to participation
   1. Logistics(e.g., travel distance. financial difficulties)
   2. Illness
   3. Embarrassment/shame
8. Response of treatment team
9. Suggestion for future improvements
10. Any other relevant factors perceived by the patient as relevant/important to attendance.
